# Incidence, distribution, seasonality, and demographic risk factors of *Salmonella* Enteritidis human infections in Ontario, Canada, 2007–2009

**DOI:** 10.1186/1471-2334-13-212

**Published:** 2013-05-10

**Authors:** Csaba Varga, David L Pearl, Scott A McEwen, Jan M Sargeant, Frank Pollari, Michele T Guerin

**Affiliations:** 1 Department of Population Medicine, Ontario Veterinary College, University of Guelph, Guelph, ON, N1G 2W1, Canada; 2 Ontario Ministry of Agriculture, Food and Rural Affairs, Guelph, ON, N1G 4Y2, Canada; 3 Centre for Public Health and Zoonoses, Ontario Veterinary College, University of Guelph, Guelph, ON, N1G 2W1, Canada; 4 Centre for Foodborne, Environmental and Zoonotic Infectious Diseases, Public Health Agency of Canada, Guelph, ON, N1H 8J1, Canada

**Keywords:** *Salmonella* enteritidis, Incidence, Poisson regression, Mixed model, Direct standardization, Demographic risk factors, Ontario, Canada

## Abstract

**Background:**

In Canada, surveillance systems have highlighted the increasing trend of *Salmonella enterica* serovar Enteritidis (*S*. Enteritidis) human infections. Our study objectives were to evaluate the epidemiology of *S*. Enteritidis infections in Ontario using surveillance data from January 1, 2007 through December 31, 2009.

**Methods:**

Annual age-and-sex-adjusted incidence rates (IRs), annual and mean age-adjusted sex-specific IRs, and mean age-and-sex-adjusted IRs by public health unit (PHU), were calculated for laboratory-confirmed *S*. Enteritidis cases across Ontario using direct standardization. Multivariable Poisson regression with PHU as a random effect was used to estimate incidence rate ratios (IRRs) of *S*. Enteritidis infections among years, seasons, age groups, and sexes.

**Results:**

The annual age-and-sex-adjusted IR per 100,000 person-years was 4.4 [95% CI 4.0-4.7] in 2007, and 5.2 [95% CI 4.8-5.6] in both 2008 and 2009. The annual age-adjusted sex-specific IRs per 100,000 person-years ranged from 4.5 to 5.5 for females and 4.2 to 5.2 for males. The mean age-adjusted sex-specific IR was 5.1 [95% CI 4.8-5.4] for females and 4.8 [95% CI 4.5-5.1] for males. High mean age-and-sex-adjusted IRs (6.001-8.10) were identified in three western PHUs, one northern PHU, and in the City of Toronto. Regression results showed a higher IRR of *S*. Enteritidis infections in 2009 [IRR = 1.18, 95% CI 1.06-1.32; P = 0.003] and 2008 [IRR = 1.17, 95% CI 1.05-1.31; P = 0.005] compared to 2007. Compared to the fall season, a higher IRR of *S*. Enteritidis infections was observed in the spring [IRR = 1.14, 95% CI 1.01-1.29; P = 0.040]. Children 0–4 years of age (reference category), followed by children 5–9 years of age [IRR = 0.64, 95% CI 0.52-0.78; P < 0.001] had the highest IRRs. Adults ≥ 60 years of age and 40–49 years of age [IRR = 0.31, 95% CI 0.26-0.37; P < 0.001] had the lowest IRRs.

**Conclusions:**

The study findings suggest that there was an increase in the incidence of *S*. Enteritidis infections in Ontario from 2007 to 2008–2009, and indicate seasonal, demographic, and regional differences, which warrant further public health attention.

## Background

Salmonellosis remains an important public health issue worldwide [1-3], causing considerable health costs [4-7] and financial losses to all members of the food supply chain [2]. Globally, non-typhoidal salmonellae (NTS) cause an estimated 93.8 million human infections and 155,000 deaths annually [3]. Non-typhoidal salmonellae are the second most frequently reported enteric bacterial pathogens in Canada [8,9], the United States of America (US) [10], and Europe [11]; and they are the top foodborne bacteria causing hospitalization and death in Canada [12] and the US [13,14].

*Salmonella enterica* serovar Enteritidis (*S*. Enteritidis) recently became the most common serotype among the NTS in the US [10], with a significantly increased incidence in 2009 compared with the periods 2006–2008 and 1996–1998 [6,14]. Moreover, in Canada, surveillance systems have highlighted the increasing trend of *S*. Enteritidis human infections, such that *S*. Enteritidis has become the most prevalent NTS serotype [8,9,15]. Considering the under-reporting rate of salmonellosis in Canada (an estimated 13 to 37 cases are unreported per reported case), the burden of infections is even higher [16].

The epidemiology of human *S*. Enteritidis infections is complex due to the multitude of risk factors that could be associated with illness. Previous epidemiological studies have revealed the following individual-level risk factors for *S*. Enteritidis infections: eating chicken outside of the home [17,18]; eating breaded, stuffed chicken products [19] and raw or undercooked eggs [18,20,21]; another infected person in the home [22]; eating food prepared by an infected food handler [23-26]; contact with birds and reptiles [26]; international travel [18,26-28]; young age [29,30]; and exposures during June and July [31]; although other risk factors might also be important.

In Canada, health regions are administrative zones demarcated by provincial ministries of health according to provincial legislations [32]. In Ontario, Canada there are 36 public health units (PHUs) that oversee health promotion and disease prevention programs. In Ontario, salmonellosis is a reportable disease under provincial legislation and all laboratory-confirmed cases are reported to the local PHU; personnel at each PHU are required to perform case investigations and enter their findings into the Ontario Ministry of Health and Long-Term Care’s (MOHLTC) integrated Public Health Information System (iPHIS) surveillance database. In addition, all clinical *Salmonella* isolates are sent to Public Health Ontario’s Toronto Public Health Laboratories for confirmation and serotyping using conventional methods [33].

Although passive surveillance systems represent an underestimation of disease burden, they provide invaluable data on enteric disease incidence and trends [34,35]. There is a need to better understand the demographic, geographic, and seasonal factors associated with the increase in human *S*. Enteritidis infections in Ontario and to provide evidence-based information for policy makers to prioritize future efforts in addressing the increasing number of infections. Thus, the objectives of this study were to 1) describe annual age-and-sex-adjusted incidence rates (IRs), and annual and mean age-adjusted sex-specific IRs of *S*. Enteritidis cases in Ontario; 2) describe the mean age-and-sex-adjusted IR for each PHU; and 3) identify associations between *S*. Enteritidis IRs and demographic and seasonal factors.

## Methods

### Data sources

In Ontario, a confirmed case of salmonellosis is defined as the isolation of *Salmonella* (excluding *Salmonella* Typhi or Paratyphi) from an appropriate clinical sample (e.g., stool, urine, blood) with or without clinically compatible signs and symptoms [36]. Data pertaining to the *S*. Enteritidis cases’ age, sex, reporting PHU, and date of illness onset were acquired from the iPHIS database. The University of Guelph Ethics Review Board was consulted because our research involved human participants; however, ethics approval was not required because our data did not contain any personal or health information that could be linked back to the original identifiers. The data represent all cases of *S*. Enteritidis that were captured within the database between January 1, 2007 and December 31, 2009. Travel-related (i.e., those who had traveled outside of Canada within 3 days before the onset of illness) and outbreaks (two or more epidemiologically-linked cases) were included in the analysis because the study objectives were to describe the overall epidemiology of *S*. Enteritidis infections in Ontario.

The Census of Canada is administered every five years by Statistics Canada, to collect demographic and socioeconomic information on Canadians [37]. Estimates based on the 2006 Census of Population for each year, age category, sex, and PHU were obtained from Statistics Canada, Demography Division [38].

### Statistical methods

The distribution of values was examined, missing data and improbable values were identified, and the data were corrected wherever possible or eliminated from the analysis. Descriptive and statistical analyses were performed using Microsoft Excel 2000 (Microsoft Corporation, Redmond, WA, USA) and STATA Intercooled statistical software, version 10.1 (Stata Corporation, College Station, TX, USA).

Direct standardization [39-41] was used with the PHU-, year-, age-, and sex-based population as the reference population to calculate annual age-and-sex-adjusted IRs, and annual and mean age-adjusted sex-specific IRs for *S*. Enteritidis cases in Ontario, and the mean age-and-sex-adjusted IR for each PHU.

**Figure 1:**
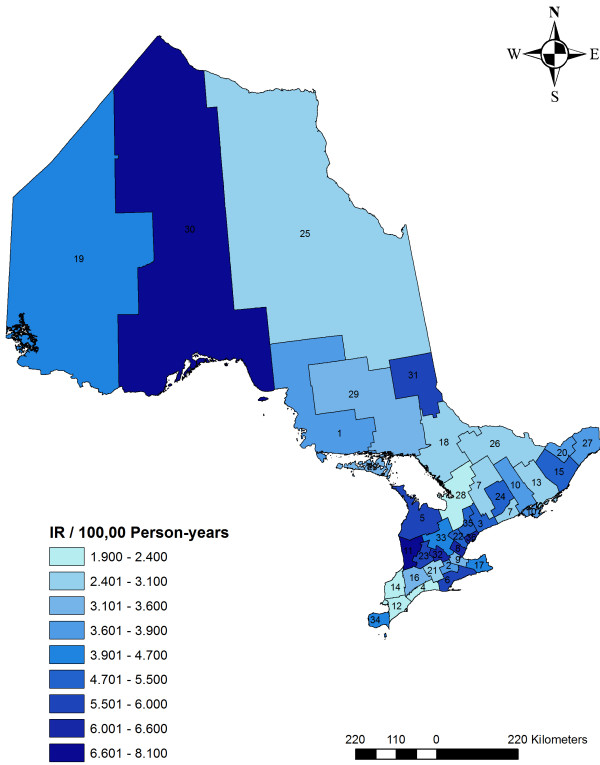
**Mean age**-**and**-**sex**-**adjusted incidence rates of *Salmonella *Enteritidis infections, across Ontario public health units, 2007-2009 **^**a)**^**. **^a)^IR- Incidence rate. Public health unit labels and names are presented in Additional file 2 – Legend 2.

To identify associations between *S*. Enteritidis IRs in Ontario and demographic and seasonal factors, a multivariable Poisson regression analysis was conducted. The dependent variable was the number of *S*. Enteritidis cases by year, season, age group, sex, and PHU (see Additional file 1 - Legend 1 for an example of the data structure). The categorical independent variables were year, season, age group, sex, and PHU. The variable PHU represented the 36 PHUs in Ontario. The PHU was included as a fixed effect because of the observed variability of the IRs across PHUs (Figure 1). The District of Algoma Health Unit, because it had the lowest IR, was used as the reference category to which the other PHUs were compared. The date of onset of illness reported by each *S*. Enteritidis case was used to assign the case to a particular year and season. When the date of onset was missing, the date when the sample was received by the laboratory or when the case was reported into the iPHIS database was used. Season was categorized as winter (December, January, and February), spring (March, April, and May), summer (June, July, and August), and fall (September, October, and November). The variable year was defined as a consecutive 12-month period from January 1^st^ to December 31^st^; thus, there were three categories for year (2007, 2008, and 2009). The variable age included ten-year age categories, with the exception of children < 4 years of age and those 5–9 years of age, which were retained because of their biological importance [42,43], and adults 60 years of age and older, which were pooled into one category because of the small number of cases in this age group. Pair-wise correlation coefficients using the Spearman’s rank test among all variables were examined. If the independent variables were highly correlated (Spearman’s rho > 0.70), variables with the smallest p-value were considered for the model building process. To address the differences in year-, age group-, sex-, and PHU-based population size estimates, we used the natural log-transformed population estimates as the offset, which accounted for the denominator when calculating incidence rate ratios (IRRs). An IRR was the IR in the category of interest compared to the IR in the reference category. Variables with a p-value equal to or less than 0.05 were considered significant and were kept in the model. Incidence rate ratios and their corresponding 95% confidence intervals were estimated. Interaction terms were created between each independent variable and tested for significance. If the interaction term was significant (p ≤ 0.05) it was retained in the final model. The model was evaluated by identifying influential observations (i.e. large values of Cook’s distance) and outliers (i.e. large values of Pearson, deviance, or Anscombe residuals) using residual plots. The overall fit of the model was assessed using Deviance and Pearson *χ*^2^ goodness-of-fit tests [44].

To account for lack of fit, a multi-level mixed-effects Poisson regression model was then constructed using the xtmepoisson command in STATA [45], which uses adaptive Gaussian quadrature to approximate the log likelihood. The model included the same dependent and independent (year, season, age group, and sex) variables as the first model with the exception that PHU was included as a random intercept instead of a fixed effect. The structure of the multi-level model included an offset representing the natural log-transformed year-, age group-, sex-, and PHU-based population size estimates. As part of assessing model fit, we examined the normality of the best linear unbiased predictors (BLUPs) [46]. Outlier and influential observations were assessed using residual plots. Bayesian information criterion (BIC) was used to compare the fit of the two models.

ArcGIS 10 (Environmental Systems Research Institute, Inc., Redlands, CA, USA) was used to create a choropleth map for mean age-and-sex-adjusted IRs across Ontario’s PHUs; Jenk's optimization classification method [47] was employed for defining the critical intervals. This method arranges data into classes based on their distribution by using an algorithm that reduces variance within groups and maximizes variance between groups.

## Results

### Descriptive statistics and direct standardized incidence rates

Between January 1, 2007 and December 31, 2009, 1,935 *S*. Enteritidis cases were reported into iPHIS in Ontario. Three cases were excluded because they lacked age or sex information. The date of onset of illness was reported for 1,670 (86.4%) cases; 230 (11.9%) and 32 (1.7 %) cases only had information on the date when the sample was received by the laboratory and the date when the case was reported into the iPHIS database, respectively. The iPHIS collects all reportable diseases throughout Ontario’s PHUs, and no major changes in salmonellosis reporting requirements or testing protocols were noted during the study period, which makes our data robust and reliable. Information on specimen type was not available; however, based on our working experience at the MOHLTC, and the literature, the majority of specimens were stool samples. No major outbreaks were declared during the study period.

The age of cases ranged from < 1 year to > 90 years. Children < 4 years of age and adults ≥ 60 years of age represented 13.1% and 13.6% of cases, respectively, while adults 20–29 years of age represented 16.8% of cases. Overall, 51.6% and 48.4% of cases were females and males, respectively.

The annual age-and-sex-adjusted IR per 100,000 person-years was 4.4 [95% CI 4.0-4.7] in 2007, and 5.2 [95% CI 4.8-5.6] in both 2008 and 2009 (Table 1). Over the study period, the annual age-adjusted sex-specific IR per 100,000 person-years ranged between 4.5 and 5.5 for females and between 4.2 and 5.2 for males (Table 1). The mean age-adjusted sex-specific IR per 100,000 person-years was 5.1 [95% CI 4.8-5.4] for females and 4.8 [95% CI 4.5-5.1] for males (Table 1).

**Table 1:** **Direct standardized incidence rates of *Salmonella *Enteritidis infections in Ontario**, **2007**–**2009** (**n** = **1**,**932 cases**)

**Year **^**a)**^		**N **^**b)**^	**IR **^**c)**^	**95 % CI **^**d)**^
2007 ^e)^		12,792,937	4.4	4.0-4.7
2008 ^e)^		13,070,584	5.2	4.8-5.6
2009 ^e)^		13,064,900	5.2	4.9-5.6
2007 ^f)^	Female	6,480,556	4.5	4.0-5.1
	Male	6,312,381	4.2	3.7-4.7
2008 ^f)^	Female	6,565,166	5.2	4.7-5.8
	Male	6,505,418	5.2	4.7-5.8
2009 ^f)^	Female	6,625,568	5.5	5.0-6.1
	Male	6,439,332	5.0	4.5-5.6
2007-2009 ^g)^	Female	19,671,290	5.1	4.8-5.4
	Male	19,257,131	4.8	4.5-5.1

^a)^ Year: A consecutive 12-month period from January 1^st^ to December 31^st^.

^b)^ N: Reference population estimates obtained from the 2006 Census of Canada.

^c)^ IR: Direct standardized incidence rate per 100,000 person-years. Denominator included year-, age-, sex-, and public health unit-based population estimates.

^d)^ CI: Confidence interval of the adjusted IR.

^e)^ Annual age-and-sex-adjusted IR.

^f)^ Annual age-adjusted sex-specific IR.

^g)^ Mean age-adjusted sex-specific IR.

**Figure 2:**
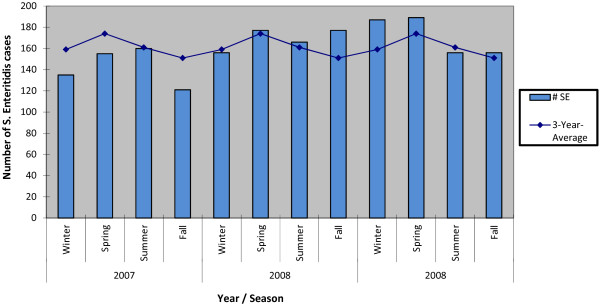
**Seasonal distribution of *Salmonella *Enteritidis (*S. *Enteritidis) cases in Ontario, 2007–2009 (n = 1,932) **^**a**)^**. **^a)^ Winter (December, January, and February), spring (March, April, and May), summer (June, July, and August), and fall (September, October, and November). Three-year-average: number of *S*. Enteritidis cases for each season divided by the number of years.

**Figure 3:**
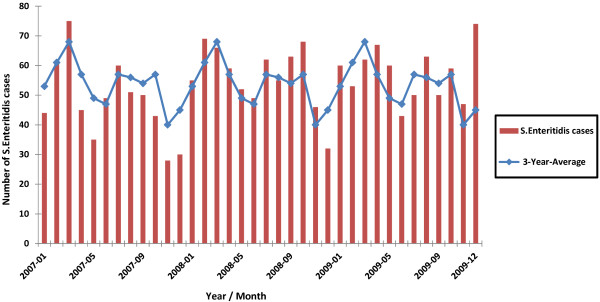
**Number of *Salmonella *Enteritidis (*S. *Enteritidis) cases by month in Ontario, 2007–2009 (n = 1,932) **^**a)**^**. **^a)^ Three-year-average: number of *Salmonella* Enteritidis cases for each month divided by the number of years.

Seasonal counts ranged from 135 to 187 cases in winter (mean over 3-year period = 160 cases), 155 to 189 in spring (mean = 173), 156 to 166 in summer (mean = 160), and 121 to 177 in fall (mean = 151) (Figure 2). The highest monthly count was 75 cases in March 2007 and the lowest was 28 cases in November 2007 (Figure 3).

The mean age-and-sex-adjusted IRs for the entire study period across Ontario’s PHUs ranged from 1.9 to 8.1 (Figure 1; Additional file 2 - Legend 2). Visually exploring the map, the highest IRs (> 6.0 per 100,000 person-years) were observed in three south-western PHUs (Halton Regional Health Unit, Huron County Health Unit, and Waterloo Health Unit), one northern PHU (Thunder Bay District Health Unit), and in the City of Toronto Health Unit.

### Poisson regression

The Deviance and Pearson *χ*^2^ goodness-of-fit test statistics for the Poisson model were 6,212.6 (P = 1.00) and 17,727.6 (P = 0.004), respectively. Several outlier and influential observations were identified; however, re-running the model without these observations did not change any of the coefficients. The BIC for the model was 9,906.4.

Because one of the two goodness-of-fit tests for the Poisson model indicated lack of fit, we used a multi-level model. No outlier or influential observations were identified for the upper level residuals of the multi-level model. The BLUPs for the PHU random intercept were normally distributed. The BIC for the multi-level model was 9,655.2, indicating a better fit.

The results of the multi-level model are shown in Table 2. Significantly higher IRRs of *S*. Enteritidis infections were reported in 2009 [IRR = 1.18, 95% CI 1.06-1.32] and 2008 [IRR = 1.17, 95% CI 1.05-1.31] compared to 2007. Compared to the fall season, a significantly higher IRR of *S*. Enteritidis infections was reported in the spring [IRR = 1.14, 95% CI 1.01-1.29]. Children 0–4 years of age (reference category), followed by children 5–9 years of age [IRR = 0.64, 95% CI 0.52-0.78] had the highest IRRs of infection. Adults ≥ 60 years of age and 40–49 years of age [IRR = 0.31, 95% CI 0.26-0.37] had the lowest IRRs of infection. No statistically significant difference in *S*. Enteritidis infection rates were detected between sexes.

**Table 2:** **Risk factors for *Salmonella *Enteritidis infections in humans**, **Ontario**, **Canada**, **2007**–**2009** (**n** = **1**,**932 cases**)

**Variable **^**a)**^		**IRR **^**b)**^	**95% CI **^**c)**^	**P-value **^**d)**^
Year	2007	Reference	-	-
	2008	1.17	1.05-1.31	0.005
	2009	1.18	1.06-1.32	0.003
Season	Fall	Reference	-	-
	Spring	1.14	1.01-1.29	0.040
	Summer	1.06	0.93-1.20	0.377
	Winter	1.06	0.93-1.20	0.413
Age (years)	0-4	Reference	-	-
	5-9	0.64	0.52-0.78	< 0.001
	10-19	0.44	0.34-0.51	< 0.001
	20-29	0.51	0.43-0.60	< 0.001
	30-39	0.34	0.28-0.40	< 0.001
	40-49	0.31	0.26-0.37	< 0.001
	50-59	0.33	0.28-0.40	< 0.001
	≥ 60	0.31	0.26-0.37	< 0.001
Sex	Female	Reference	-	-
	Male	0.95	0.87-1.04	0.273
Intercept		0.00002	0.00002-0.00003	< 0.001

^a)^ Multi-level mixed-effects Poisson regression model using adaptive Gaussian quadrature. Public health unit (PHU) was included as a random intercept. Variance of PHU random effects = 0.074 [95% CI = 0.036-0.153]. Dependent variable: number of *Salmonella* Enteritidis cases by year, season, age group, sex, and PHU. Offset: natural log-transformed year-, age group-, sex-, and PHU-based population size estimates. ^b)^ IRR: Incidence rate ratio for categorical independent variables, in which the incidence rate (IR) in the category of interest was compared to the IR in the reference category. ^c)^ CI: Confidence interval of the IRR. ^d)^ Statistically significant at P ≤ 0.05.

## Discussion

Our study is the most current and geographically diverse investigation from Ontario, and fills information gaps related to current knowledge of the incidence, demographic determinants, distribution, and seasonality of human *S*. Enteritidis infections.

From 2007 to 2008–2009, an increase in annual age-and-sex-adjusted IRs of *S*. Enteritidis infections was identified. Moreover, the Poisson regression model revealed a significantly higher IRRs of *S*. Enteritidis infections in 2008 and 2009 compared to 2007. This finding is in agreement with the results of current Canadian [15] and US [6] surveillance that have shown an increase in *S*. Enteritidis infections. *Salmonella* Enteritidis continues to be a key cause of human enteric illness and poses a substantial health burden to the North American population [3]. Reducing the incidence of *S*. Enteritidis infections is challenging due to the variety of transmission routes and contaminated food sources [26], the possible increase in environmental reservoir(s), and changes in food processing and safety practices [48]; however, increased efforts should be directed toward mitigation strategies for this pathogen.

Our study demonstrated that young children 0–4 years of age had the highest *S*. Enteritidis infection IRRs, which is in agreement with results of other studies from developed countries [29,30,42,49,50]. Previous studies identified several risk factors for *S*. Enteritidis infections for this age group, including international travel [51,52], riding in shopping carts and exposure to raw meat and poultry products [53], and contact with reptiles [43,52,54] and cats [43]. In our study, adults 60 years of age and older had the lowest IRR among all age groups, which is in contrast with other studies [29,55]. This finding was unexpected because typically the two age group extremes have the highest rates of enteric infections. Prospective research studies are needed in Ontario to assess differences in *S*. Enteritidis infection rates between age groups that are attributed to various exposures.

Examination of seasonal differences in *S*. Enteritidis rates in our study revealed a higher IRR of infections during the spring (March through May). The higher incidence in spring might be associated with international travel. Travel has been identified as an important risk factor for *S*. Enteritidis, and it was shown in recent Ontario studies that a large proportion of *S*. Enteritidis cases, especially in the winter and spring, were travel-related [28,56].

We did not find a statistically significant difference in the IRR of *S*. Enteritidis infections between females and males, which is consistent with a previous US study [29].

When analyzing the differences in the incidence rates of *S*. Enteritidis infections among Ontario’s PHUs, we calculated mean age-and-sex-adjusted IRs using direct standardization. This method is useful when the prevalence of exposures might differ among age groups, sexes, and PHUs. We used geographic information system software to create choropleth maps for IRs of *S*. Enteritidis infections across PHUs in Ontario. This is a useful technique to visualize the findings of conventional statistical analysis, and by using Jenk's optimization classification for defining the critical intervals for mapping the IRs, it allowed us to identify high risk PHUs. Future research studies should be conducted to identify and assess novel transmission routes, spatio-temporal trends, and socioeconomic status indicators that might have an impact on the emergence of *S*. Enteritidis infections in these regions.

Before extrapolating our results to the whole Ontario population, a few limitations need to be noted. It is essential to mention that laboratory surveillance systems generally underestimate the true burden of enteric diseases in a population for several reasons. There might be differences in underreporting across age groups, because children and older adults are more likely to visit a physician, and physicians are more likely to request stool samples from them for testing. Moreover, there might be geographic variation in underreporting of *S*. Enteritidis infections due to differences in health care providers’ accessibility, and in the sensitivity of laboratory methods used at different laboratories [34,35,57]. Finally, misclassification of cases might have occurred when cases were categorized into year and season. However this bias was likely minor because the majority of cases had date of illness onset (or date of sample reception) information. The difference between date of illness onset and the date when the samples were received by the laboratory could be estimated to be a maximum of one week, considering the time of delivery of samples within Ontario, and the incubation period of *S*. Enteritidis that ranges from half to three days [58].

## Conclusions

Our results showed higher IRRs of *S*. Enteritidis infections in 2008 and 2009 compared to 2007, and indicate seasonal and regional differences, with a higher IRR of *S*. Enteritidis infections in the spring. In Ontario, we found that children 0–4 years of age were at the highest risk for *S*. Enteritidis infections. These results provide evidence-based information that will assist policy makers to prioritize future efforts in addressing the increase in the number of *S*. Enteritidis infections in the human population in Ontario. We recommend that children, and PHUs with high *S*. Enteritidis rates, be targeted for prevention and control programs designed to decrease the incidence of *S*. Enteritidis. Further case–control and ecological studies are needed to identify novel risk factors (food sources, socioeconomic determinants, and transmission routes) and spatio-temporal trends for *S*. Enteritidis infections in Ontario.

## Abbreviations

BIC: Bayesian information criterion; BLUP: best linear unbiased predictor; iPHIS: integrated Public Health Information System; IR: incidence rate; IRR: incidence rate ratio; MOHLTC: Ontario Ministry of Health and Long-Term Care; NTS: non-typhoidal salmonellae; PHU: Public Health Unit; S. Enteritidis: *Salmonella enterica* serovar Enteritidis; US: United States of America.

## Competing interests

The authors declare that they have no competing interests.

## Authors’ contributions

CV developed the study design, analyzed the data, interpreted results, wrote the first draft of the manuscript, responded to editorial comments, and prepared the final manuscript for submission. MTG and DLP were consulted for study design, data analysis, and interpretation of results, and reviewed and commented on manuscript drafts. SAM, FP and JMS provided advice on data analysis, interpretation of results, and reviewed and commented on manuscript drafts. All authors read and approved the final manuscript.

## Pre-publication history

The pre-publication history for this paper can be accessed here:

http://www.biomedcentral.com/1471-2334/13/212/prepub

## Supplementary Material

Additional file 1 **Legend 1.** Example of data structure. Click here for file

Additional file 2 **Legend 2 for Figure** 1**.** Ontario Public Health Units labels and names. Click here for file
