# Supplementary material for: Incidence, distribution, seasonality, and demographic risk factors of Salmonella Enteritidis human infections in Ontario, Canada, 2007–2009
Source: BMC Infect Dis. 2013 May 10;13:212. doi: 10.1186/1471-2334-13-212 (PMC3655886; doi:10.1186/1471-2334-13-212)
Supplement: Additional file 1 — Legend 1. Example of data structure. [file 1471-2334-13-212-S1.pdf]

**Number of cases**	**Year**	**Season**	**Age-range**	**Gender**	**Public Health Unit**	**Population Estimates**
0	2007	Fall	30-34	Female	City of Toronto Health Unit	108866
0	2007	Spring	30-34	Female	City of Toronto Health Unit	108866
2	2007	Summer	30-34	Female	City of Toronto Health Unit	108866
1	2007	Winter	30-34	Female	City of Toronto Health Unit	108866
1	2007	Fall	30-34	Male	City of Toronto Health Unit	104869
1	2007	Spring	30-34	Male	City of Toronto Health Unit	104869
4	2007	Summer	30-34	Male	City of Toronto Health Unit	104869
0	2007	Winter	30-34	Male	City of Toronto Health Unit	104869
1	2008	Fall	30-34	Female	Waterloo Health Unit	18384
0	2008	Spring	30-34	Female	Waterloo Health Unit	18384
0	2008	Summer	30-34	Female	Waterloo Health Unit	18384
0	2008	Winter	30-34	Female	Waterloo Health Unit	18384
0	2008	Fall	30-34	Male	Waterloo Health Unit	19080
1	2008	Spring	30-34	Male	Waterloo Health Unit	19080
0	2008	Summer	30-34	Male	Waterloo Health Unit	19080
0	2008	Winter	30-34	Male	Waterloo Health Unit	19080
0	2009	Fall	30-34	Female	Waterloo Health Unit	18432
1	2009	Spring	30-34	Female	Waterloo Health Unit	18432
0	2009	Summer	30-34	Female	Waterloo Health Unit	18432
1	2009	Winter	30-34	Female	Waterloo Health Unit	18432
0	2009	Fall	30-34	Male	Waterloo Health Unit	18950
1	2009	Spring	30-34	Male	Waterloo Health Unit	18950
1	2009	Summer	30-34	Male	Waterloo Health Unit	18950
0	2009	Winter	30-34	Male	Waterloo Health Unit	18950
1	2008	Fall	55-59	Female	YorkRegional Health Unit	30605
1	2008	Spring	55-59	Female	YorkRegional Health Unit	30605
0	2008	Summer	55-59	Female	YorkRegional Health Unit	30605
2	2008	Winter	55-59	Female	YorkRegional Health Unit	30605
0	2008	Fall	55-59	Male	YorkRegional Health Unit	30007
0	2008	Spring	55-59	Male	YorkRegional Health Unit	30007
0	2008	Summer	55-59	Male	YorkRegional Health Unit	30007
0	2008	Winter	55-59	Male	YorkRegional Health Unit	30007
