# Supplementary material for: Incidence, distribution, seasonality, and demographic risk factors of Salmonella Enteritidis human infections in Ontario, Canada, 2007–2009
Source: BMC Infect Dis. 2013 May 10;13:212. doi: 10.1186/1471-2334-13-212 (PMC3655886; doi:10.1186/1471-2334-13-212)
Supplement: Additional file 2 — Legend 2 for Figure 1. Ontario Public Health Units labels and names. [file 1471-2334-13-212-S2.pdf]

1	The District of Algoma Health Unit	19	Northwestern Health Unit
2	Brant County Health Unit	20	City of Ottawa Health Unit
3	Durham Regional Health Unit	21	Oxford County Health Unit
4	Elgin-St. Thomas Health Unit	22	Peel Regional Health Unit
5	Grey Bruce Health Unit	23	Perth District Health Unit
6	Haldimand-Norfolk Health Unit	24	Peterborough County-City Health Unit
7	Haliburton, Kawartha, Pine Ridge District Health Unit	25	Porcupine Health Unit
8	Halton Regional Health Unit	26	Renfrew County and District Health Unit
9	City of Hamilton Health Unit	27	The Eastern Ontario Health Unit
10	Hastings and Prince Edward Counties Health Unit	28	Simcoe Muskoka District Health Unit
11	Huron County Health Unit	29	Sudbury and District Health Unit
12	Chatham-Kent Health Unit	30	Thunder Bay District Health Unit
13	Kingston, Frontenac, and Lennox and Addington Health Unit	31	Timiskaming Health Unit
14	Lambton Health Unit	32	Waterloo Health Unit

15	Leeds, Grenville and Lanark District Health Unit	33	Wellington-Dufferin-Guelph Health Unit
16	Middlesex-London Health Unit	34	Windsor-Essex County Health Unit
17	Niagara Regional Area Health Unit	35	York Regional Health Unit
18	North Bay Parry Sound District Health Unit	36	City of Toronto Health Unit
